# Association Between Treated Periodontal Disease and Febrile Neutropenia in Perioperative Chemotherapy for Breast Cancer: A Retrospective Cohort Study

**DOI:** 10.7759/cureus.51349

**Published:** 2023-12-30

**Authors:** Ai Yamaguchi, Yuki Kataoka, Kazuma Fujimura, Tomoe Taji, Hirofumi Suwa

**Affiliations:** 1 Department of Breast Surgery, Hyogo Prefectural Amagasaki General Medical Center, Amagasaki, JPN; 2 Section of Clinical Epidemiology, Department of Community Medicine, Kyoto University Graduate School of Medicine, Kyoto, JPN; 3 Department of Healthcare Epidemiology, Kyoto University Graduate School of Medicine/School of Public Health, Kyoto, JPN; 4 Department of Systematic Reviewers, Scientific Research Works Peer Support Group (SRWS-PSG), Kyoto, JPN; 5 Department of Internal Medicine, Kyoto Min-iren Asukai Hospital, Kyoto, JPN; 6 Department of Oral and Maxillofacial Surgery, Hyogo Prefectural Amagasaki General Medical Center, Amagasaki, JPN

**Keywords:** retrospective cohort study, perioperative chemotherapy, febrile neutropenia, breast cancer, periodontal disease

## Abstract

Background: This study aimed to examine whether the incidence of febrile neutropenia (FN) during perioperative chemotherapy for breast cancer increased in patients with periodontal disease who had received prior dental treatment.

Methods: This retrospective cohort study conducted at a single tertiary care center included patients diagnosed with clinical stages I-III of breast cancer and had started neoadjuvant or adjuvant intravenous chemotherapy between July 2015 and November 2021. The exposure was periodontal disease (probing depth ≥6 mm) diagnosed by dentists before the start of chemotherapy. Almost all the patients received dental treatment and oral care before initiating chemotherapy. The primary outcome was FN incidence during chemotherapy. We used a multivariable logistic regression model adjusted for age, diabetes mellitus, chemotherapy regimen, and the mean relative dose intensity.

Results: Based on the eligibility criteria of this study, 141 women were included. The incidence of FN in the periodontal group (probing depth ≥6 mm) and control group (probing depth <6 mm) was 36.4% and 25.9%, respectively. The crude odds ratio (OR) for FN incidence was 1.63 (95% confidence interval [CI], 0.71-3.74; P = 0.24), and the adjusted OR was 1.52 (95% CI, 0.62-3.73; P = 0.36).

Conclusions: Occurrence of FN during perioperative chemotherapy for breast cancer is not a concern in patients undergoing dental treatment for periodontal disease before or during chemotherapy.

## Introduction

Periodontal disease not only causes tooth loss but also has systemic effects. It is a risk factor for pneumonia [1], and it could affect the prognosis of diabetes negatively [2]. The Community Periodontal Index (CPI) is a screening method for assessing periodontal status, which uses the World Health Organization (WHO) probe to evaluate periodontal pockets, gingival bleeding, and calculus [3]. The prevalence of periodontal disease is quite high. In the Japanese general population, the proportion of individuals with periodontal pockets ≥4 mm in depth increased with age, with approximately 45% and 60% of the individuals in their 40s and 60s, respectively. The proportion of those with gingival bleeding was >30% among individuals aged ≥15 years and >40% in those aged 30-55 years [4].

Moreover, gingivitis and periodontitis are a concern in patients with neutropenia undergoing chemotherapy. Microorganisms can migrate from the ulcerated periodontal pocket epithelium into the bloodstream [5]. Bacterial products, such as lipopolysaccharides, and pro-inflammatory cytokines in the inflamed and infected periodontal lesions may induce a cascade of systemic inflammatory responses [6,7]. In fact, a retrospective study of hospitalized adults with leukemia demonstrated that patients with gingivitis/periodontitis had a higher risk of sepsis (27.8% vs. 19.6%), bacterial infections (19.5% vs. 10.1%), and fungal infections (20.7% vs. 10.7%) than those without [8]. However, to date, no studies on solid tumors have been reported, which are usually treated with less intense myelosuppressive chemotherapy than hematopoietic malignancies.

This study examined whether the incidence of febrile neutropenia (FN) increases in patients with periodontal disease during perioperative chemotherapy for breast cancer.

## Materials and methods

Study design

This retrospective cohort study was conducted at a single tertiary care center. It was approved by the Ethics Committee of our institution (2-144) and was conducted in accordance with the Code of Ethics of the World Medical Association (Declaration of Helsinki). The duration of the study is from November 2020 to May 2022. Complete information about the study was made available to the participants, who were given the opportunity to refuse participation.

We identified patients for this study using chemotherapy databases and retrieved their clinical information, including clinical findings, dental records, chemotherapy regimen, tumor type, and toxicity data, from the hospital’s electronic medical records. The STROBE checklist [9] followed is provided as an online resource [10].

Participants

The inclusion criteria for this study were age ≥20 years, histological diagnosis of invasive breast cancer clinical stages I-III according to the Union for International Cancer Control [11], and start of neoadjuvant or adjuvant intravenous chemotherapy between July 2015 and November 2021. Patients who had not consulted our Dental Surgery Department before chemotherapy, those without probing depth data during dental examination, and those with edentulous jaws were excluded.

Chemotherapy regimens

The chemotherapy regimen was determined according to the Japanese Breast Cancer Society Clinical Practice Guidelines [12]. The regimen was selected at the discretion of the clinician if there were concerns regarding toxicity or contraindications to a particular drug. Most cases received sequential administration of four cycles of epirubicin 90 mg/m^2^ plus cyclophosphamide 600 mg/m^2^ (EC) every 21 days and four cycles of docetaxel (DTX) 75 mg/m^2^ every 21 days (or 12 cycles of paclitaxel [PTX] 80 mg/m^2^ every seven days). In human epidermal growth factor receptor type 2-positive cases, trastuzumab or trastuzumab plus pertuzumab was administered concurrently with taxane. Granulocyte colony-stimulating factor (G-CSF) was administered prophylactically with four to six cycles of DTX 75 mg/m^2^ plus cyclophosphamide 600 mg/m^2^ every 21 days (TC) and dose-dense chemotherapy (four cycles of EC every 14 days administered sequentially with four cycles of PTX 175 mg/m^2^ every 14 days or 12 cycles of PTX 80 mg/m^2^ every seven days). G-CSF was administered in other regimens at the discretion of the clinician. There was no concomitant use of other therapies during intravenous chemotherapy.

Exposure

The exposure was periodontal disease, and the presence or absence of the exposure was assessed by dentists before starting chemotherapy. The probing depth around all the teeth was evaluated using the WHO probe. It was defined as the distance from the gingival margin to the base of the pocket and expressed in millimeters; periodontal disease was defined when the probing depth was ≥6 mm, according to the CPI recommended by the WHO [3].

The radiographic alveolar bone loss was evaluated by a dentist using panoramic radiography to determine whether it extended to the middle or apical third of the root (≥Stage III according to the American Academy of Periodontology and the European Federation of Periodontology staging system [13]).

Based on complete or partial pre-cancer chemotherapy dental treatment protocols [14], personalized dental treatment was administered before starting chemotherapy. Routine oral care was provided during chemotherapy, including dental scaling, professional tooth cleaning, extraction of infected teeth, and tooth brushing instructions.

Primary outcome

The primary outcome was the incidence of FN during intravenous chemotherapy. The observation period was defined as that from the start to the end of chemotherapy.

The onset of FN was defined as antibiotic administration at some point between the day after starting chemotherapy and the last day of chemotherapy. Antibiotics were limited to those used for FN at our institution, i.e., ciprofloxacin, amoxicillin-clavulanate, levofloxacin, meropenem, cefepime, piperacillin, tazobactam, and vancomycin. We excluded patients with no episodes of axillary temperature ≥37.5°C documented in the medical records before or after the date of antibiotic administration.

As a countermeasure against FN, oral antibiotics (ciprofloxacin plus amoxicillin-clavulanate or levofloxacin) were administered at the start of chemotherapy. We instructed patients to take oral antibiotics without visiting our institution if they recorded an axillary temperature ≥37.5°C after the start of chemotherapy. If the patient was asymptomatic and the fever resolved within a few days of receiving oral antibiotics, there was no need to visit our institution.

Secondary outcome

The secondary outcomes were a mean relative dose intensity (RDI) <85%, percentage of delayed days for chemotherapy (≥15 days), and incidence of hematologic adverse events ≥Grade 2 according to the National Cancer Institute Common Terminology Criteria for Adverse Events, version 5.0. The RDI was calculated as follows: (actual dose [mg/m^2^/week] / standard dose [mg/m^2^/week]) × 100 [15].

Statistical analysis

Univariate and multivariate analyses were performed using logistic regression to determine the odds ratio (OR) and 95% confidence interval (CI). The confounders considered were age, diabetes, chemotherapy regimen, and the RDI. Model 1 was adjusted for age, diabetes mellitus (glycated hemoglobin [HbA1c] ≥6.5% or <6.5%), and probing depth (≥6 mm or <6 mm). Model 2 was adjusted for age, diabetes mellitus (HbA1c ≥6.5% or <6.5%), probing depth (≥6 mm or <6 mm), non-anthracycline-based chemotherapy regimen (yes or no), and RDI (<85% or ≥85%). Two-sided probability testing was used; P-values <0.05 were considered statistically significant. Statistical analyses were performed using EZR 1.55 software package (R statistical software version 4.1.2, R Foundation for Statistical Computing, Vienna, Austria) [16].

## Results

**Figure 1:**
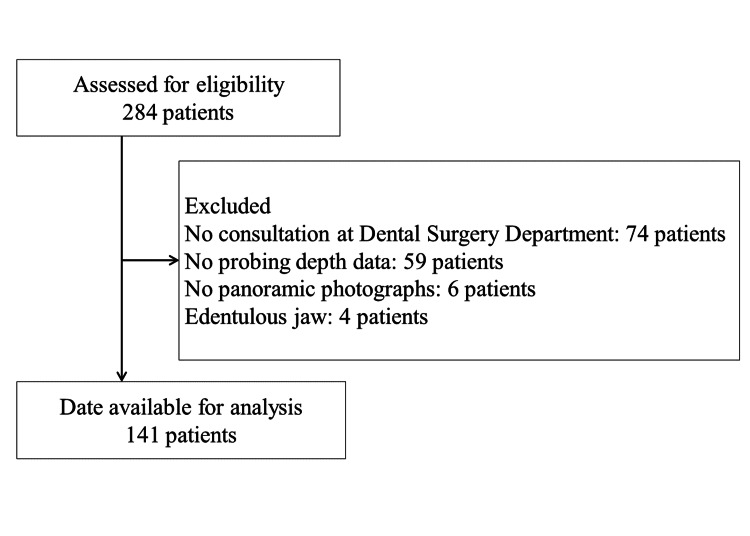
Patient flow chart

In total, 141 patients were included in the analysis (Figure 1). The observation period lasted until May 15, 2022, when all patients in the study had completed chemotherapy. There were no dropouts during the observation period. Baseline patient performance status was 0 or 1, absolute neutrophil count ≥1500/µL, total bilirubin <2 mg/dL, aspartate aminotransferase (AST) <100 U/L, alanine aminotransferase (ALT) <100 U/L, and creatinine <1.5 mg/dL. (Only one patient had an ALT of 138 U/L and received a reduced dose.) Table 1 shows the baseline clinicopathological characteristics of the patients.

**Table 1:** Baseline clinicopathologic characteristics of patients (N=141) ER, estrogen receptor; PR, progesterone receptor; HER2, human epidermal growth factor receptor type 2; NAC, neoadjuvant chemotherapy; EC epirubicin plus cyclophosphamide; AC, doxorubicin plus cyclophosphamide; FEC, 5-fluorouracil, epirubicin plus cyclophosphamide, DTX, docetaxel; PTX, paclitaxel; Nab-PTX, nanoparticle albumin-bound paclitaxel; TC, docetaxel plus cyclophosphamide; TCb, docetaxel plus carboplatin; Tra, trastuzumab; Per, pertuzumab; G-CSF, granulocyte colony-stimulating factor; HbA1c, hemoglobin A1c; RABL, radiographic alveolar bone loss.

Variable	All patients (N =141)	Probing depth (mm)	
		≥ 6 (N =33)	< 6 (N =108)
Age, median (range)	55 (25-77)	61 (41-77)	54 (25-76)
Postmenopausal, n (%)	85 (60.3)	24 (72.7)	61 (56.5)
Histology, n (%)			
Invasive ductal	130 (92.2)	30 (90.9)	100 (92.6)
Invasive lobular	4 (2.8)	1 (3.0)	3 (2.8)
Other	5 (3.5)	2 (6.1)	3 (2.8)
Missing data	2 (1.4)	0 (0)	2 (1.9)
Histological grade, n (%)			
Grade 1	28 (19.9)	7 (21.2)	21 (19.4)
Grade 2	87 (61.7)	20 (60.6)	67 (62.0)
Grade 3	21 (14.9)	3 (9.1)	18 (16.7)
Missing data	5 (3.5)	3 (9.1)	2 (1.9)
Clinical stage, n (%)			
Stage 1	31 (22.0)	12 (36.4)	19 (17.6)
Stage 2	86 (61.0)	16 (48.5)	70 (64.8)
Stage 3	22 (15.6)	4 (12.1)	18 (16.7)
Missing data	2 (1.4)	1 (3.0)	1 (0.9)
ER positive, n (%)	85 (60.3)	20 (60.6)	65 (60.2)
PR positive, n (%)	74 (52.5)	16 (48.5)	58 (53.7)
HER2 positive, n (%)	45 (31.9)	11 (33.3)	34 (31.5)
NAC, n (%)	57 (40.4)	11 (33.3)	46 (42.6)
Chemotherapy regimen, n (%)			
Taxane plus anthracycline-based	108 (76.6)	25 (75.8)	83 (76.9)
EC or AC or FEC → DTX or PTX or Nab-PTX	96 (68.1)	21 (63.6)	75 (69.4)
Dose-dense therapy	12 (8.5)	4 (12.1)	8 (7.4)
Non-anthracycline-based	31 (22.0)	8 (24.2)	23 (21.3)
TC	16 (11.3)	1 (3.0)	15 (13.9)
TCb	4 (2.8)	1 (3.0)	3 (2.8)
DTX	1 (0.7)	0 (0)	1 (0.9)
Nab-PTX	2 (1.4)	1 (3.0)	1 (0.9)
PTX	8 (5.7)	5 (15.2)	3 (2.8)
Other	2 (1.4)	0 (0)	2 (1.9)
Tra or/plus Per	45 (31.9)	11 (33.3)	34 (31.5)
G-CSF for prophylaxis, n (%)	46 (32.6)	12 (36.4)	34 (31.5)
HbA1c ≥ 6.5%, n (%)	14 (9.9)	6 (18.2)	8 (7.4)
Hypertension, n (%)	27 (19.1)	11 (33.3)	16 (14.8)
Hyperlipidemia, n (%)	26 (18.4)	7 (21.2)	19 (17.6)
Asthma, n (%)	5 (3.5)	1 (3.0)	4 (3.7)
Hypothyroidism, n (%)	4 (2.8)	3 (9.1)	1 (0.9)
Atrial fibrillation, n (%)	3 (2.1)	0 (0)	3 (2.8)
Hyperthyroidism, n (%)	2 (1.4)	1 (3.0)	1 (0.9)
Other comorbidities, n (%)	7 (5.0)	1 (3.0)	6 (5.6)
Assessment of periodontal condition as per the community periodontal index			
Bleeding on probing, n (%)	41 (29.1)	15 (45.5)	26 (24.1)
Dental calculus, n (%)	81 (57.4)	16 (48.5)	65 (60.2)
Number of teeth, median (range)	27 (5-32)	27 (11-32)	27 (5-32)
Tooth mobility, n (%)	11 (7.8)	4 (12.1)	7 (6.5)
Periapical lesion, n (%)	21 (14.9)	7 (21.2)	14 (13.0)
RABL ≥ Stage Ⅲ, n (%)	34 (24.1)	17 (51.5)	17 (15.7)
Number of oral care visits per month, median (range)	1.1 (0-3.4)	1.1 (0-2.3)	1.1 (0-3.4)

The patients were divided into the periodontal group (probing depth ≥6 mm; n = 33) and the control group (probing depth <6 mm; n = 108), and the mean age was greater in the former than in the latter. The number of participants receiving PTX monotherapy was more in the periodontal group than in the control group. Table 2 presents the adverse events, delayed days, and mean RDI values of the two groups.

**Table 2:** Adverse events, delayed days, and relative dose intensity during chemotherapy (N=141) FN, febrile neutropenia; AST, aspartate aminotransferase; ALT, alanine aminotransferase; RDI, relative dose intensity.

Variable		All patients (N =141)	Probing depth (mm)	
			≥ 6 (N =33)	< 6 (N =108)
FN, n (%)		40 (28.4)	12 (36.4)	28 (25.9)
Hospital visit at the time of FN, n (%)		8 (5.6)	2 (6.0)	6 (5.6)
Hospitalization due to FN, n (%)		5 (3.6)	1 (3.0)	4 (3.7)
Adverse events, n (%)				
Neutropenia	Grade 2	9 (6.4)	4 (12.1)	5 (4.6)
	≥ Grade 3	106 (75.2)	23 (69.7)	83 (76.9)
Anemia	Grade 2	51 (36.2)	12 (36.4)	39 (36.1)
	≥ Grade 3	6 (4.3)	1 (3.0)	5 (4.6)
Platelet count decrease	Grade 2	4 (2.8)	1 (3.0)	3 (2.8)
	≥ Grade 3	0 (0.0)	0 (0.0)	0 (0.0)
AST increased	Grade 2	1 (0.7)	0 (0.0)	1 (0.9)
	≥ Grade 3	4 (2.8)	0 (0.0)	4 (3.7)
ALT increased	Grade 2	15 (10.6)	4 (12.1)	11 (10.2)
	≥ Grade 3	11 (7.8)	1 (3.0)	10 (9.3)
Delayed days, median (range)		7 (0-56)	5 (0-56)	7 (0-56)
Delayed days (≥ 15 days), n (%)		29 (20.6)	3 (9.1)	26 (24.1)
RDI%, median (range)		94.2 (51.6-101.8)	96.1 (51.6-100.3)	94.2 (52.8-101.8)
RDI (< 85%), n (%)		25 (17.7)	6 (18.2)	19 (17.6)

The incidence of FN was 36.4% (12/33) in the periodontal group and 25.9% (28/108) in the control group. Eight patients had a hospital visit at the time of FN, and blood cultures were collected and confirmed negative in seven patients. The crude OR for FN incidence was 1.63 (95% CI, 0.71-3.74; P = 0.24) (Table 3), and the adjusted OR was 1.52 (95% CI, 0.62-3.73; P = 0.36) (Model 2 in Table 4).

**Table 3:** Univariate logistic regression analysis of factors associated with febrile neutropenia OR, odds ratio; CI, confidence interval; G-CSF, granulocyte colony-stimulating factor, RDI, relative dose intensity; HbA1c, hemoglobin A1c.

Variable		Univariate analysis	
		OR (95% CI)	P-value
Age		1.01 (0.97-1.04)	0.56
Taxane plus anthracycline-based	No	1.00	
	Yes	2.68 (0.95-7.55)	0.061
Non-anthracycline-based	No	1.00	
	Yes	0.21 (0.06-0.74)	0.015
G-CSF for prophylaxis	No	1.00	
	Yes	0.41 (0.17-0.99)	0.048
RDI	≥ 85%	1.00	
	< 85%	0.57 (0.20-1.67)	0.31
Neutropenia	< Grade 3	1.00	
	≥ Grade 3	5.72 (1.64-19.9)	0.006
HbA1c	< 6.5%	1.00	
	≥ 6.5%	1.46 (0.45-4.66)	0.52
Probing depth	< 6 mm	1.00	
	≥ 6 mm	1.63 (0.71-3.74)	0.24
Bleeding on probing	No	1.00	
	Yes	1.26 (0.56-2.77)	0.57
Dental calculus	No	1.00	
	Yes	2.11 (0.96-4.61)	0.06
Number of teeth		0.98 (0.92-1.06)	0.75
Tooth mobility	No	1.00	
	Yes	1.49 (0.41-5.40)	0.54
Periapical lesion	No	1.00	
	Yes	0.75 (0.25-2.23)	0.61

**Table 4:** Multivariate logistic regression analysis of factors associated with febrile neutropenia OR, odds ratio; CI, confidence interval; RDI, relative dose intensity; HbA1c, hemoglobin A1c.

Variable		Multivariate analysis			
		Model 1		Model 2	
		OR (95% CI)	P-value	OR (95% CI)	P-value
Age		1.00 (0.97-1.04)	0.80	1.01 (0.97-1.05)	0.42
Non-anthracycline-based	No			1.00	
	Yes			0.20 (0.05-0.71)	0.013
RDI	≥ 85%			1.00	
	< 85%			0.53 (0.17-1.65)	0.217
HbA1c	< 6.5%	1.00		1.00	
	≥ 6.5%	1.30 (0.39-4.27)	0.66	1.30 (0.38-4.42)	0.67
Probing depth	< 6 mm	1.00		1.00	
	≥ 6 mm	1.54 (0.65-3.67)	0.32	1.52 (0.62-3.73)	0.36

## Discussion

This study compared the incidence of FN during perioperative breast cancer chemotherapy between patients with and without periodontal disease. The incidence of FN was slightly higher in the periodontal group than in the control group; however, the confidence interval for the OR straddled unity.

The risk of infection might increase due to chemotherapy-induced neutropenia, particularly in patients with periodontal disease [8,17-21]. Several studies have demonstrated the benefits of dental and periodontal treatments prior to chemotherapy. In patients with hematopoietic malignancies, dental treatment decreases the incidence of systemic infections and inflammatory complications during chemotherapy [22-24]. Almost all patients in this study received dental treatment prior to chemotherapy and oral care during chemotherapy. The risk of FN in patients with or without periodontal disease may be lesser in those who receive dental treatment and oral care than in those who do not.

The risk of FN may be relatively low in patients with periodontal disease who undergo dental treatment. The OR is not high enough to have clinical effects as compared to that of other factors for FN reported previously (TC therapy: OR, 2.67; age ≥65 years: OR, 2.24) [25]. Furthermore, the reported OR was 2.4, assuming a 30% incidence of FN in those with periodontal disease and 15% in those without periodontal disease [8,25]. This suggests that with dental care before or during chemotherapy, the development of FN in patients with periodontal disease does not have clinical effects, and patients can safely undergo chemotherapy.

The strength of this study is its novelty, as we compared the incidence of FN during chemotherapy for solid tumors in patients with and without periodontal disease; however, it has several limitations. First, it was a single-center, retrospective, observational study and may have been underpowered to detect statistically significant differences. Second, cases other than those of FN may have been included because FN was defined when antimicrobials were administered for fever, and assessing the neutrophil count was not mandatory. Third, 74 patients who did not visit the Dental Surgery Department before chemotherapy were excluded, which may have caused a bias in the results. Since dental examinations may not have been performed due to the absence of apparent dental problems, patients with periodontal disease were less likely to be excluded. Therefore, a multicenter prospective study in which all breast cancer patients undergo probing depth measurements before perioperative chemotherapy is warranted.

## Conclusions

It was suggested that the risk of FN is relatively low in patients with periodontal disease. FN incidence is not a major concern during perioperative chemotherapy in early breast cancer patients with periodontal disease if they receive regular oral care and dental treatment. The study may be underpowered to detect statistically significant differences. A prospective multicenter study is needed to evaluate the association between periodontal disease and FN in breast cancer patients undergoing preoperative chemotherapy.
